# Mutations in the H, F, or M Proteins Can Facilitate Resistance of Measles Virus to Neutralizing Human Anti-MV Sera

**DOI:** 10.1155/2014/205617

**Published:** 2014-02-04

**Authors:** Hasan Kweder, Michelle Ainouze, Sara Louise Cosby, Claude P. Muller, Camille Lévy, Els Verhoeyen, François-Loïc Cosset, Evelyne Manet, Robin Buckland

**Affiliations:** ^1^INSERM-U1111, 69007 Lyon, France; ^2^ENS-Lyon, 69007 Lyon, France; ^3^University of Lyon, UCB-Lyon1, 69007 Lyon, France; ^4^LabEx Ecofect, University of Lyon, 69007 Lyon, France; ^5^School of Medicine, Dentistry and Biomedical Sciences, Queen's University, BT7 1NN Belfast, UK; ^6^Institute of Immunology, Public Research Center for Health/LNS, 20A rue Auguste Lumière, L-1950 Luxemburg, Grand-Duchy of Luxembourg, Luxembourg

## Abstract

Although there is currently no evidence of emerging strains of measles virus (MV) that can resist neutralization by the anti-MV antibodies present in vaccinees, certain mutations in circulating wt MV strains appear to reduce the efficacy of these antibodies. Moreover, it has been hypothesized that resistance to neutralization by such antibodies could allow MV to persist. In this study, we use a novel *in vitro* system to determine the molecular basis of MV's resistance to neutralization. We find that both wild-type and laboratory strain MV variants that escape neutralization by anti-MV polyclonal sera possess multiple mutations in their H, F, and M proteins. Cytometric analysis of cells expressing viral escape mutants possessing minimal mutations and their plasmid-expressed H, F, and M proteins indicates that immune resistance is due to particular mutations that can occur in any of these three proteins that affect at distance, rather than directly, the native conformation of the MV-H globular head and hence its epitopes. A high percentage of the escape mutants contain mutations found in cases of Subacute Sclerosing Panencephalitis (SSPE) and our results could potentially shed light on the pathogenesis of this rare fatal disease.

## 1. Introduction

Measles virus is (MV), a member of the genus *Morbillivirus* in the family Paramyxoviridae. The MV virion is enveloped and contains a nonsegmented negative-strand RNA genome encoding six structural proteins: N, P, M, F, H, and L. The genome is encapsidated by the N (nucleoprotein) which is associated with the P and L proteins (viral polymerase) to form the helical ribonucleoprotein complex (RNP). The glycoproteins H and F are embedded as spikes in the virion membrane. The H protein (hemagglutinin) is responsible for attachment to the cellular receptors of MV and the F protein is responsible for the consequent fusion of the virion membrane with the host cell's plasma membrane whereby the RNP is delivered into the cytoplasm, and the matrix protein M lines the inner surface of the virion membrane [1]. In the infected cell, the glycoproteins accumulate in the plasma membrane. This allows the H protein to interact with cellular receptors on neighboring uninfected cells and cause cell-cell fusion (syncytia formation) through activation of the F protein. Moreover, in the case of the infected cell, evidence exists to suggest that the M protein interacts with the cytoplasmic tails of the glycoproteins H and F at the plasma membrane [2, 3]. As far as cellular receptors for MV are concerned, the wt strains have been shown to use Signaling Lymphocyte Activation Molecule (SLAM; CD150) whereas the vaccine and laboratory strains use both SLAM and CD46 [4]. Expression of SLAM is restricted to cells of the human immune system whereas CD46 is expressed ubiquitously. Recently, a third receptor, the epithelial adherens junction protein nectin-4, has been identified [5, 6].

MV is a serologically monotypic virus and in theory, vaccination should provide life-long protection. However, the proportion of the population possessing only vaccine-induced immunity has increased over time with reduced exposure to wild-type MV infection and there is now evidence of resistance of recent measles virus wild-type isolates to antibody-mediated neutralization in vaccinees. This includes individuals with not only primary but also secondary vaccine failure [7, 8] and is a concern for global MV elimination. It is evident that a better understanding of the molecular basis of MV's escape from neutralizing antibody is required.

In a previous study, we used mutagenesis to allow lentiviral vectors pseudotyped with MV glycoproteins to escape neutralizing antibodies. The use of such vectors in *in vivo* use has been hampered by their susceptibility to anti-MV polyclonal antibodies that are present in the sera of most humans due to extensive vaccination. As the MV-H glycoprotein appears to be the principal target for these anti-MV sera [9], we introduced mutations into the major epitopes of the MV-H globular head to try to overcome this problem [10]. Although neutralization was partially reduced by the introduction of such mutations, we were able to increase protection by adding the mutation D416N that had the effect of providing an extra glycosylation site. This mutation, which is present in modern MeV strains that appear to better resist neutralization [11], presumably restricts access of anti-MV antibodies to the major MV-H epitopes. The possibility that this change has arisen *in vivo* in response to immune pressure suggested to us that generating viral escape mutants *in vitro* could be a potential means to identify other mutations affecting neutralization. Moreover, sequencing of membrane proteins from escape mutants and subsequent construction of mutant viral proteins potentially could allow the mutation responsible for immune escape to be identified.

We thus attempted to mimic immune selection *in vivo* by putting MV under selective pressure *in vitro* using polyclonal antibody sera. Hitherto, monoclonal antibodies (mAbs) have been used for making escape mutants [12] but polyclonal sera have also been shown to contain highly prevalent amounts of conformation-dependent antibodies [13]. We used sera from both healthy MV-vaccinated donors and a Subacute Sclerosing Panencephalitis (SSPE) patient. SSPE is a rare fatal disease of children and young adults caused by wild-type MV persisting in the human brain 8–10 years after an apparently banal acute infection (see [14] for a recent review). The SSPE serum was used because such sera have been shown to contain elevated levels of anti-MV antibodies [15]. Mutants isolated using this system were sequenced to identify the mutations they contained. As the globular head of the H protein contains the binding sites for the cellular receptors of MV, we were particularly interested in identifying mutations in this protein. However, we speculated that mutations allowing immune escape do not necessarily have to reside in the MV-H globular head nor indeed within this protein. Our reasoning for this was based on the knowledge that both of the MV glycoproteins are required for fusion [16] and that they are associated physically in the endoplasmic reticulum before being transported to the plasma membrane of MV-infected cells [17]. Relatively minor conformational changes in the H protein, induced post-attachment to the cellular receptor, are believed to be transmitted to the F protein that then undergoes the major conformational changes that lead to the fusion process [18]. Moreover, it has been shown that the matrix protein (M) is associated with the cytoplasmic tails of both glycoproteins [2, 3]. Thus, the H, F, and M proteins of MV can be considered to form a trimeric protein complex in the infected cell's plasma membrane. Moreover, there is evidence that interaction of the M protein with the glycoprotein cytoplasmic tails can have an “inside-out” effect on their extracellular domains and hence their function [2, 3]. For example, recombinant MV that does not contain the M protein produces a higher rate of cell-cell fusion than the complete virus [3]. Such a requirement for the maintenance of a physical interaction between these three proteins could potentially allow mutations other than in the MV-H globular head to have an effect on MV-H epitopes.

Our results show that MV variants that escape *in vitro* immune neutralization possess multiple mutations not only in their H proteins, but also in their F and M proteins. In order to identify which mutations were allowing immune escape to occur, we focused on viral mutants with a minimal number of mutations. Cytometric analysis of cells expressing these minimal mutants and their individual H, F, and M proteins indicated that escape from neutralization by anti-MV sera can be due to mutations occurring in any of these three proteins: such mutations affect at distance, rather than directly, the native conformation of the MV-H globular head and hence its epitopes. Our results thus suggest that immune selection can occur in nature and reinforces calls for the constant monitoring of emerging wild-type MV strains. Interestingly, many of the mutations generated in our *in vitro* system are found in SSPE cases, which could suggest that immune selection potentially plays a role in SSPE pathogenesis. Although SSPE viruses—unlike our escape mutants—are nonfusogenic and hence noninfectious, it is probable that their lack of fusogenicity is due to the multiple mutations that they accumulate over several years within the human brain. To have had the capacity to cause the original infection, that later led to SSPE, the virus must have been fusogenic.

## 2. Materials and Methods

### 2.1. Cells

Vero cells and Vero/hSLAM (Vero cells constitutively expressing human SLAM) were maintained in Dulbecco's modified Eagle's medium (DMEM) supplemented with 10% fetal bovine serum (FBS), 2 mM L-glutamine, 100 U/mL penicillin, 0.1 mg/mL streptomycin, and 10 mM HEPES. Chinese hamster ovary cells constitutively expressing human SLAM (CHO/hSLAM) were maintained in F12 medium containing 10% fetal bovine serum (FBS), 100 U/mL penicillin, 0.1 mg/mL streptomycin, and 1x MEN nonessential amino acids.

### 2.2. Antibodies

Antibodies used for flow cytometry studies included an SSPE serum (from a 1978 Belfast case), anti-MV-H mAbs 55 (SLAM-binding site) [19], BH129 (anti-NE epitope) [20], BH216 (anti-Noose epitope) [21], and anti-MV-F mAb Y503 (a gift from D. Gerlier, Inserm U758). Secondary antibodies were rabbit anti-human IgG and goat anti-mouse IgG (both from Millipore). For the western blot an anti-MV-H mAb BH195 [20] and an anti-MV-F mAb (a gift from Christian Buchholz, Paul Ehrlich Institute, Langen, Germany) were used together with secondary antibodies, polyclonal rabbit anti-mouse immunoglobulins/HRP, and polyclonal goat anti-rabbit immunoglobulins/HRP, respectively (Dako). Antibodies used for the confocal microscope studies were anti-MV-H mAb BH129, anti-MV-F mAb Y503, and anti-MV-M mAb 8910 (Millipore).

### 2.3. Viruses and Production of Escape Mutants

Both a wild-type MV strain (G954) and a laboratory strain (Halle) were used to obtain the escape mutants. We used six different sera from healthy persons immunized against MV and one serum from a SSPE patient. Sera were heated at 56°C for 45 minutes to inactivate complement. In general, cells were infected by MV in the presence of low concentrations of serum. Medium was changed every 2 days, and then the virus was plaque purified in the presence of an anti-MV serum. The serum concentration was then increased gradually over 1-2 months of passaging. Subsequently, after 8–10 passages, two selection steps were made to isolate escape mutant clones. Vero/hSLAM cells were used to obtain SLAM-dependent escape mutants of the wild-type strain; Vero cells were used to obtain CD46-dependant escape mutants of the Halle strain and CHO/hSLAM to obtain SLAM-dependant escape mutants of the Halle strain. Escape mutants were amplified, and the viral titers were calculated.

### 2.4. Viral Amplification and Titration

A virus stock was made following amplification: cells in 2 mL of medium were frozen at −80°C overnight 2-3 days after infection when most cells showed fusion/syncytium formation. The medium was then thawed and harvested and the virus stock titrated. Cells in 96-well tissue culture plates were inoculated with 1/10 serially diluted culture medium samples for 1 h at 37°C. The inocula were then removed and new medium added to each well. After 4 days, the number of infected wells was counted and the number of plaque-forming units (PFU) calculated.

### 2.5. RT-PCR: Subcloning of Hemagglutinin (H), Fusion (F), and Matrix (M) Genes and Sequencing

Vero/hSLAM cells in 6-well tissue culture plates were infected with Halle and G954 escape mutants at a multiplicity of infection (m.o.i) of 0.01 for 1 h at 37°C using a virus stock diluted in 1 mL of nonsupplemented DMEM. Infection media were then removed and fresh medium was added. 36 h after infection an extraction of total RNA was made using the RNeasy kit (QIAGEN) and then RT-PCR and subcloning of H, F, and M genes into the phCMV plasmid were performed. All of the final constructs were fully sequenced.

### 2.6. Fusion Quantification

To study fusion, Vero cells or CHO/hSLAM (CD46-expressing or SLAM-expressing, resp.) according to the mutant tested were infected in 6-well plates by the different escape mutants + (nonmutated) Halle at a m.o.i of 0.01. Fusion was quantified 30–36 h after-infection as described previously [22].

### 2.7. Transient Neutralization Assay

The standard Plaque Reduction Neutralization Test (PRNT_50_) was used: the concentration of serum that reduces the plaque number by at least 50% gives a measure of its neutralizing capacity—the PRNT_50_ value. For each of the mutants + (nonmutated) Halle, serial two-fold dilutions of sera were mixed with the same volume of virus and incubated for 90 minutes at 37°C. The mixtures were then placed on Vero cells (at 80% confluence) at 37°C, 5% CO_2_, for 60 minutes. The supernatant was then discarded and 30% carboxymethyl cellulose (CMC) was added to reduce the spread of viral particles. The number of syncytia/plaques was counted after 4 days of incubation: to visualize the plaques, the CMC was washed 1x with PBS and then 0.1% crystal violet solution was added.

### 2.8. Introduction of Mutations into the MeV-H, MeV-F, and MeV-M Genes Expressed From Plasmid PhCMV

Some escape mutants in this study have more than one mutation in their H, F, or M genes. To determine the effect of these mutations separately, mutations were introduced separately in the different genes using the QuickChange mutagenesis kit (Stratagene) according to the manufacturer's instructions. These mutations were introduced in the genes encoding the H, F, and M proteins cloned into the plasmid phCMV. The mutated plasmids were amplified and purified. All mutations were verified by DNA sequencing.

### 2.9. Transfection

Cells were transfected using jetPRIME (short protocol; Polyplus). For the flow cytometry, cells in 6-well plates were transfected using 2 *μ*g of phCMV-H or 2 *μ*g of phCMV-F for transfection of single plasmids. For double and triple transfections, 1 *μ*g of phCMV-H, 1 *μ*g of phCMV-F and 0.5 *μ*g of phCMV-M were used. After 4 h incubation at 37°C, the medium was changed for medium containing the fusion inhibitor peptide (FIP) [23] to prevent cell-cell fusion. For transfections with a single plasmid, there was no requirement to change the medium or to use FIP. For the western blot assay, cells in 100 mm culture vessel were transfected using 10 *μ*g of either phCMV-H or phCMV-F.

### 2.10. Viral Infection

For the flow cytometry, cells in 6-well plates were infected at a m.o.i of 0.2 for 1 h at 37°C using a virus stock diluted in 1 mL of nonsupplemented DMEM. Infection media were then removed and fresh medium containing FIP was added. For western blot assay, 75 cm^2^ flasks were used.

### 2.11. Flow Cytometry

24 h after infection or transfection, cells were prepared for flow cytometry analysis. Generally, four sera were used as primary antibodies with the following concentrations: 1/2000 SSPE serum, 1/10 mAb 55 (anti-H), 1/100 mAb BH129 (anti-H), and 1/6000 mAb 503 (anti-F). Alternatively, 1/100 mAb BH216 (anti-H) was used.

### 2.12. Western Blot Assay

24 h after infection or transfection, surface proteins were biotinylated then extracted, using the Pierce Cell Surface Protein Isolation Kit. Total protein was also extracted from the same samples. The proteins were separated using SDS-PAGE and then transferred to an Immobilon-P membrane (Millipore). To reveal the H and F proteins, 1/5000 mAb BH195 and 1/2000 mAb CB were used as primary antibodies, respectively.

### 2.13. Confocal Microscope Study for the Localization of MV Proteins, H, F, and M

Vero-SLAM cells, grown on glass cover slides in 12-well culture plates, were cotransfected with 0.5 µg phCMV-H, 0.5 µg phCMV-F, and 0.5 µg phCMV-M using jetPRIME (short protocol; Polyplus). Cells were subjected to immunofluorescence 24 h post-transfection. Three antibodies were used: anti-H mAb BH129, anti-F mAb Y503, and anti-M mAb8910 (Millipore). Antibodies were labelled using Zenon Mouse IgG Labeling Kits (Molecular Probes). Anti-H mAb BH129 was stained with Alexa Fluor 488, anti-F mAb Y503 with Alexa Fluor 555, and anti-M mAb8910 with Alexa Fluor 647. Cells were first washed with PBS 1x. Then live cells were incubated only with stained anti-H and anti-F for 1 h at 4°C. Next, cells were washed with PBS, fixed with 3% PFA, and permeabilized with 0.1% Triton X-100 for 10 minutes at RT. Subsequently, cells were washed with blocking solution (0.2% Tween 20, 2% BSA, 5% Glycerol in PBS) and incubated in blocking solution for 10 minutes. Cells were incubated with labelled anti-M for 1 h at 4°C and then the slides were prepared for confocal microscope study. Laser argon, laser 561, and laser 633 were used for H, F, and M respectively. The specimens were studied in two steps, H and M in one step and F in another step to avoid interference between the emission signals of H, F, and M.

## 3. Results

### 3.1. Wild-Type MV Escape Mutants Selected *In Vitro* with Anti-MV and SSPE Patient Polyclonal Sera Contain Multiple Mutations Distributed between Their H, F, and M Proteins

The wild-type (wt) MV escape mutants obtained with our *in vitro* selection system are listed in Table 1. These were obtained by putting the G954 strain of MV under selective pressure *in vitro* using polyclonal antibody sera from both healthy MV-vaccinated donors and an SSPE patient. SLAM-dependant escape mutants were selected using Vero-SLAM cells. Following selection, the H, F, and M genes of each escape mutant were sequenced in order to identify mutations. The escape mutants are listed in Table 1 according to the serum (from six MV vaccinees or an SSPE patient) used for their selection. Table 1 shows that the majority of wt MV escape mutants obtained with the *in vitro* selection system contain multiple mutations that are spread within the different domains of the two glycoproteins and the matrix protein. Surprisingly, it was observed that 12 of the 14 escape mutants (86%) contain at least one “SSPE mutation” (in bold in Table 1)—that is, a mutation that has also been found in an SSPE case. 42% of the H proteins, 57% of the M proteins, and 36% of the F proteins from the escape mutants contain at least one “SSPE mutation.”

Assuming that the viral variants escape neutralization by anti-MV antibodies principally via alterations in glycoprotein epitopes, we intended to make flow cytometry studies in order to identify those that manifested a reduction in relative fluorescence intensity in interaction with any of our anti-MV sera or antibodies. However, the presence of SSPE mutations in our wt MV escape mutants alerted us to the potential danger of their manipulation. We therefore repeated this experiment using the vaccine/laboratory MV strain Halle which is nonvirulent in man.

### 3.2. Vaccine Strain MV Escape Mutants Selected *In Vitro* with Anti-MV and SSPE Patient Polyclonal Sera Also Contain Multiple Mutations Distributed between Their H, F, and M Proteins

The Halle strain MV escape mutants obtained with our *in vitro* selection system are listed in Table 2. As with the wt G954 MV strain these were obtained by putting the Halle strain of MV under selective pressure *in vitro* using polyclonal antibody sera from both healthy MV-vaccinated donors and an SSPE patient. Table 2 shows that the majority of escape mutants obtained with the *in vitro* selection system contain multiple mutations that are again spread within the different domains of the two glycoproteins and the matrix protein. As was observed in the wt MV strain, a high percentage contained mutations that are found in SSPE cases: 31 of the 42 escape mutants (74%) contain at least one “SSPE mutation” (in bold in Table 2); 43% of the H proteins, 36% of the M proteins, and 21% of the F proteins from the escape mutants contain at least one “SSPE mutation.”

To investigate whether the escape mutants possessed a reduced capacity to interact with anti-MV sera or antibodies, we next made flow cytometry studies in the presence of the antifusion peptide FIP [23]. As Table 2 indicates, 74% of the escape mutants demonstrated a reduction in relative fluorescence intensity or “negative shift” (cytometric data not shown). However, the problem of identifying the particular mutations responsible remained. To simplify this task, six escape mutants that caused a shift and contained a minimal number of mutations were selected (Table 2) for further characterization.

### 3.3. Study of the Capacities of the Selected Escape Mutants to Provoke Cell-Cell Fusion and to Resist Neutralization by Anti-MV Sera

In order to evaluate the fitness of the six mutants selected, their capacity to induce the fusion relative to (nonmutated) Halle was evaluated as well as their capacity to resist neutralization by the anti-MV sera.

All of the mutants have the capacity to provoke cell-cell fusion because this allowed their isolation. However, we wanted to investigate how their capacities to induce fusion compared with (nonmutated) Halle virus. We previously showed that both glycoproteins are required in order for fusion to occur [16]; moreover, it has been shown that the fusion provoked by a particular MV is indirectly related to the strength of the interaction between the H and F glycoproteins [24]. The results (summarized in Table 3) show that fusion induced by five out of the six mutants was lower than that induced by Halle, suggesting that there is a tighter association between the two glycoproteins in these mutants. One mutant (Halle-SLAM 2.3) was shown to induce an elevated level of fusion compared to Halle, suggesting that in this case the two glycoproteins are more loosely associated.

We also employed a transient neutralization test to investigate whether the mutants were capable of resisting neutralization at increased concentrations of the anti-MV sera. The results, summarized in Table 4, show that this was indeed the case for all six mutants. It should be noted, however, that although the mutants we have isolated are, following convention, called “escape mutants,” the adjective “escape” refers to their elevated resistance to anti-MV sera rather than an absolute capacity to escape neutralization.

We next investigated which of the mutations in each mutant was responsible for conferring the capacity to better resist neutralization.

### 3.4. Evidence of Immune Escape Resulting from a Mutation in the H Globular Head That Is Not in a Major Epitope

#### 3.4.1. Escape Mutant Halle-SLAM 1.2

**Figure 1:**
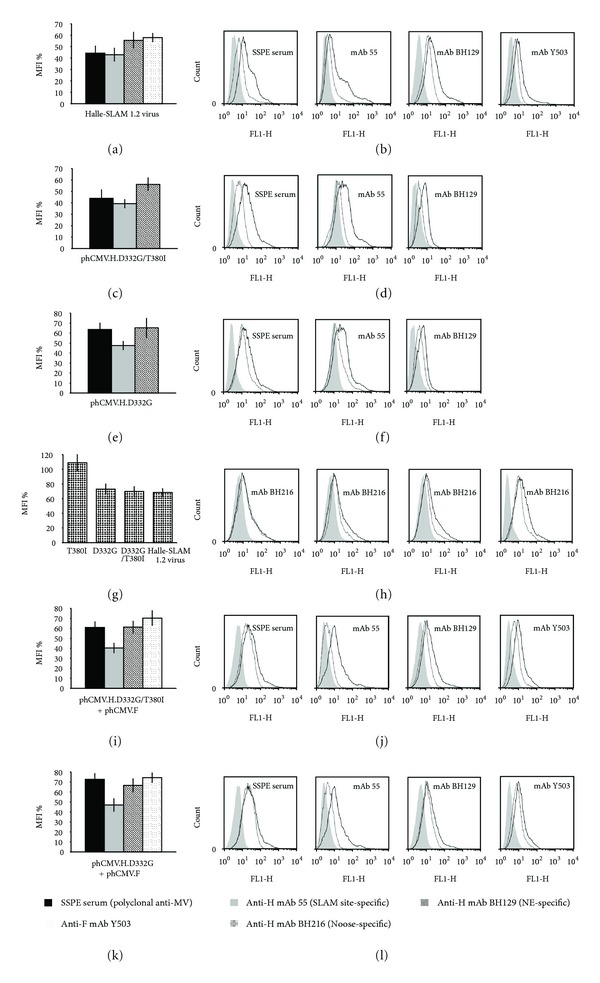
Flow cytometry analysis of escape mutant Halle-SLAM 1.2 (H: D332G, T380I; F: no change; M: Y114H, I319T). Vero-SLAM cells infected with the escape mutant virus or transfected with phCMV plasmids expressing the mutated H protein, either alone or with a phCMV plasmid expressing the nonmutated F protein, in the presence of antifusion tripeptide FIP were analysed 1 day after infection (or after transfection) using the indicated antibodies. Each overlay histogram plot represents one representative experiment; the MFI histogram data represent the mean percentages ± standard deviations for three such experiments. These values were obtained by using nonmutated Halle virus or expressed nonmutated Halle proteins as controls. (a) and (b) escape mutant Halle-SLAM 1.2-infected cells; ((c) and (d)) and ((e) and (f)), cells transfected with phCMV.H.D332G/T380I and phCMV.H.D332G, respectively; (g) and (h) cells transfected with different phCMV.H mutants or infected with the escape mutant; ((i) and (j)) and ((k), (l)), cells cotransfected with phCMV.F + phCMV.H.D332G/T380I or phCMV.H.D332G, respectively.

DNA sequencing showed that this SLAM-dependant escape mutant has two mutations in the H protein (D332G, T380I), two in the M protein (Y114H, I319T), but none in the F protein. It should be noted that one of the H protein mutations (T380I) localizes to the Noose epitope [21] on the globular head. The cytometric analysis of Vero-SLAM cells infected by this escape mutant revealed important reductions in the relative fluorescence intensity for the interaction with the polyclonal SSPE serum and the anti-SLAM binding site mAb 55 (56% and 57%, resp.; *P* < 0.001), and more modest reductions for the anti-H NE epitope mAb BH129 and the anti-F mAb Y503 (44% and 42%, resp.; *P* < 0.005) (Figures 1(a) and 1(b)).

To investigate the contribution of the mutated H protein alone we transfected Vero-SLAM cells with the expression plasmid phCMV, expressing the mutated H protein (phCMV.H.D332G/T380I), and the cytometric analysis again showed significant decreases in the SSPE serum and mAb 55 (56% and 61%, resp.; *P* < 0.001) and a more modest reduction (44%; *P* < 0.005) in mAb BH129 (Figures 1(c) and 1(d)). The two mutations were then tested separately and surprisingly we found that D332G (Figures 1(e) and 1(f)) rather than T380I plays the essential role for the observed negative shifts (data not shown). As the mutation T380I localizes to the Noose epitope, we compared the Halle-SLAM 1.2 escape mutant virus and the mutated H proteins expressed from phCMV.H.D332G/T380I, phCMV.H.D332G, and phCMV.H.T380I for their capacity to be stained by the anti-Noose antibody, mAb BH216 [21]. All these constructions induced reductions in the relative fluorescence intensity except phCMV.H.T380I (Figures 1(g) and 1(h)).

Interestingly, although no mutations are present in the F protein of this mutant, a shift was observed with anti-F mAb Y503 (Figures 1(a) and 1(b)). We thus cotransfected phCMV.H.D332G/T380I, phCMV.H.D332G, or phCMV.H.T380I with the phCMV.F protein to investigate a possible influence on the latter. The subsequent cytometric analysis showed that the D332G mutation (compare (i,j) with (k,l) in Figure 1), not T380I (data not shown), is responsible for the shift with mAb Y503. It should be noted that although there are slight differences between the negative shifts obtained with phCMV.H.D332G/T380I and phCMV.H.D332G, they are not significant statistically. To determine whether the mutated M protein plays a role in the shifts observed, we cotransfected cells with phCMV.H + phCMV.F + phCMV.M.Y114H/I319T but no such shifts were observed with any of the antibodies (data not shown). Moreover, co-transfection of cells with phCMV.H.D332G/T380I + phCMV.F + either phCMV.M or phCMV.M.Y114H/I319T indicated that the two mutations in the M protein did not play a role in the observed shifts (data not shown).

**Figure 2:**
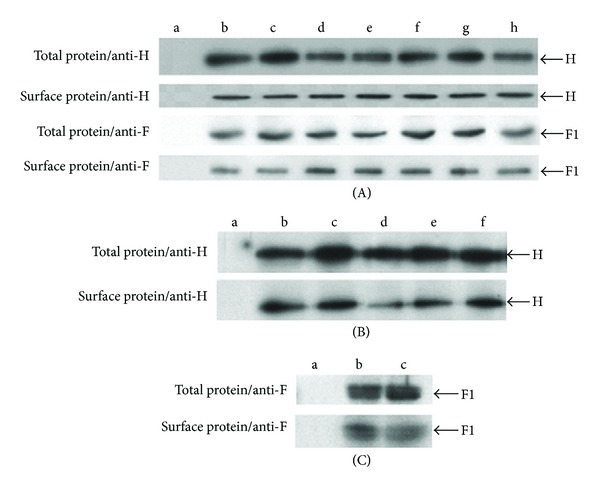
Determination of cell surface expression of escape mutant glycoproteins. Vero-SLAM cells infected with the escape mutant viruses or transfected with phCMV plasmids expressing the mutated H or mutated F proteins in the presence, when necessary, of the antifusion tripeptide FIP were lysed 1 day after infection (or after transfection) with or without previous biotinylation to extract the surface proteins or the total proteins, respectively. Western blot analysis was then done to detect the H and F proteins using anti-H mAb BH195 and anti-F mAb CB. (A) Cells infected with escape mutant viruses: (a) noninfected cells, (b) normal Halle, (c) Halle-CD46 1.2, (d) Halle-CD46 1.3, (e) Halle-CD46 3.3, (f) Halle-SLAM 1.2, (g) Halle-SLAM 2.3, and (h) Halle-SLAM 5.1. (B) Cells transfected with phCMV plasmids expressing H proteins of escape mutants: (a) normal Halle-F (control), (b) normal Halle-H, (c) Halle-CD46 1.3 H, (d) Halle-CD46 3.3 H, (e) Halle-SLAM 1.2 H, and (f) Halle-SLAM 2.3 H. (C) Western blot to detect the expression of Halle-SLAM 5.1 F; (a) normal Halle-H, (b) normal Halle-F, and (c) Halle-SLAM 5.1 F.

To eliminate the possibility that the reductions in the relative fluorescence intensity of the glycoproteins are due to lowered cellular transport we systematically used a biotinylation/western blot assay to investigate their cell surface expression for each escape mutant studied. The results for escape mutant Halle-SLAM 1.2, suggest that both the nonmutated F protein and the mutated H protein of this escape mutant are present at the cell surface in amounts similar to those obtained for the wt virus (Figure 2(A), f). A similar result was obtained for the mutated H protein expressed from plasmid phCMV (Figure 2(B), e).

These results suggest that a mutation at distance, but still within the same domain of the H protein, can be more important for changing the conformation of an epitope—and thus changing its interaction with an antibody—than a mutation that touches the epitope directly.

### 3.5. Evidence That Immune Escape Can Result from Mutations in the MeV-H Stalk Region and Globular Head Acting in Concert

#### 3.5.1. Escape Mutant Halle-CD46 1.3

**Figure 3:**
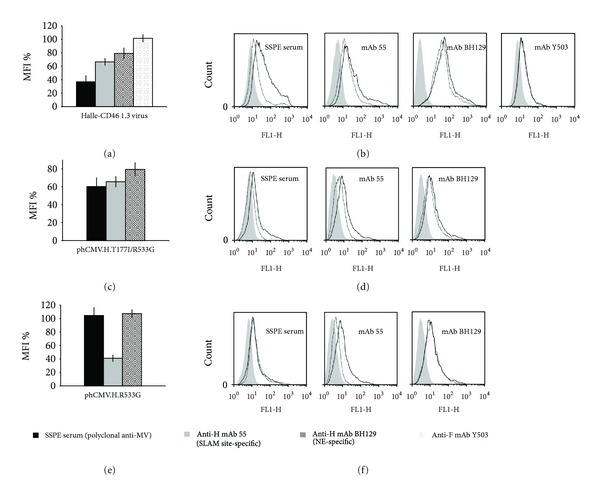
Flow cytometry analysis of escape mutant Halle-CD46 1.3 (H: T177I, R533G; F: no change; M: I69T, I196V). Vero-SLAM cells infected with the escape mutant virus or transfected with phCMV plasmid expressing the mutated H, in the presence of antifusion tri-peptide FIP when necessary, were analysed 1 day after infection (or after transfection) using the indicated antibodies. Each overlay histogram represents one experiment; the MFI histogram data represent the mean percentages ± standard deviations for three experiments. These values were obtained by using nonmutated Halle virus or expressed nonmutated Halle-H protein as controls. (a) and (b)) Escape mutant Halle-CD46 1.3-infected cells; ((c) and (d)) and ((e) and (f)) cells transfected with phCMV.H.T177I/R533G and phCMV.H.R533G, respectively.

This CD46-dependant escape mutant has two mutations in the H protein (T177I and R533G), two in the M protein (I69T and I196V), but none in the F protein. The H protein mutations occur in separate domains: the stalk (T177I) and the globular head (R533G). It should be noted that this latter mutation occurs in a residue important for the SLAM-binding site [19, 25]. Indeed, the cytometric analysis for Vero-SLAM cells infected with this escape mutant shows a reduced interaction with the SSPE serum and with mAb 55 (63%; *P* < 0.001 and 33%; *P* < 0.05 resp.) and while there is a reduction in mAb BH129, it is not statistically significant (Figures 3(a) and 3(b)). Very similar results were obtained with cells transfected with phCMV.H.T177I/R533G (Figures 3(c) and 3(d)) that suggest that the mutated H protein alone is responsible for the immune escape. However, the observed reduction in the relative fluorescence intensity with SSPE serum was more important with the escape mutant virus than with its H protein (63% in comparison with 40%, resp.; *P* for the difference: < 0.05). We thus transfected Vero-SLAM cells with either phCMV.H.T177I or phCMV.H.R533G. We found that with the former construction there was no reduction in the interaction with SSPE serum or mAb 55 (data not shown). Importantly, although in the latter case the mutation R533G largely reduced the interaction with mAb 55 (59%), there was no concomitant negative shift with the SSPE serum (see Figures 3(e) and 3(f)). Our interpretation of these results is that although the R533G mutation greatly perturbs the mAb 55 epitope—residue R533 is an important component of the SLAM binding-site [19, 25]—the presence of both mutations perturbs other epitopes in the H protein, which is reflected by the negative shift with the (anti-MeV polyclonal) SSPE serum. As the negative shift observed with phCMV.H.T177I/R533G + phCMV.F + phCMV.M.I69T/I196V was similar to that obtained with phCMV.H.T177I/R533G and with phCMV.H.T177I/R533G + phCMV.F and no such shift was observed with phCMV.H + phCMV.F + phCMV.M.I69T/I196V (data not shown), we concluded that the mutations present in the M protein of this escape mutant play no role in its escape. Moreover, the biotinylation/western blot assay demonstrated that the mutated H protein and nonmutated F protein in this escape mutant are well expressed at the cell surface (Figure 2(A), d). A similar result was obtained for the mutated H protein expressed from plasmid phCMV (Figure 2(B), c). It should be noted that the mutation T177I is present in a SSPE strain, UK85/56 (accession number: AF399850).

Importantly, the results for this escape mutant suggest that mutations in one domain of the H protein, the stalk, can cause conformational changes in another domain, the globular head, that result in a loss of epitope recognition.

### 3.6. Evidence That a Single Mutation in the MeV-H Stalk Region Can Be Sufficient to Allow Immune Escape

#### 3.6.1. Escape Mutant Halle-SLAM 2.3

**Figure 4:**
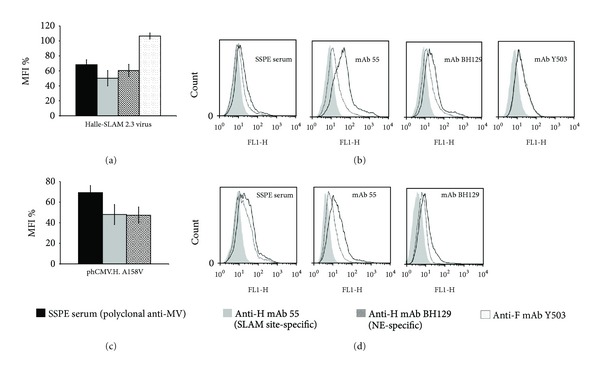
Flow cytometry analysis of escape mutant Halle-SLAM 2.3 (H: A158V; F: no change; M: M239I). Vero-SLAM cells infected with the escape mutant virus or transfected with phCMV expressing the mutated H, in the presence of antifusion tripeptide FIP when necessary, were analysed 1 day after infection or after transfection using the four antibodies indicated. Each overlay histogram represents one experiment; the MFI histogram data represent the mean percentages ± standard deviations for three experiments. These values were obtained by using nonmutated Halle virus or expressed nonmutated Halle-H protein as controls. (a) and (b)) Escape mutant Halle-SLAM 2.3-infected cells; ((c) and (d)) cells transfected with phCMV.H.A158V.

This SLAM-dependant escape mutant has one mutation in the H protein (A158V) that localizes to the stalk region, one mutation in the M protein (M239I), and no mutations in the F protein. For the Vero-SLAM cells infected with this escape mutant, the cytometric analysis revealed important reductions in the interaction with anti-H mAbs 55 and BH129 (50% and 39%, resp.; *P* < 0.001) and a moderate reduction with SSPE serum (32%; *P* < 0.05), but no reduction occurred in the relative fluorescence intensity with anti-F mAb Y503 (Figures 4(a) and 4(b)). We then transfected Vero-SLAM cells with phCMV.H.A158V to determine whether this profile could be reproduced by the expression of the H protein alone and found that this was indeed the case (Figures 4(c) and 4(d)). In addition, co-transfection of plasmids phCMV.H.A158V + phCMV.F gave a similar profile as well as plasmids phCMV.H.A158V + phCMV.F + phCMV.M.M239I. (data not shown). Moreover, as the co-transfection of plasmids phCMV.H + phCMV.F + phCMV.M.M239I did not result in a negative shift with any of the antibodies, we concluded that the M protein mutation does not play a role in the immune escape (data not shown). Importantly, the biotinylation/western blot assay demonstrated that the mutated H protein and nonmutated F protein in this escape mutant are well expressed at the cell surface (Figure 2(A), g). A similar result was obtained for the mutated H protein expressed from plasmid phCMV (Figure 2(B), f).

The results for the Halle-SLAM 2.3 mutant suggest that a single mutation (A158V) in the stalk of the H protein can be sufficient to change the oligomeric conformational state of the H protein, the globular head, thereby perturbing the recognition of major epitopes and allowing immune escape.

### 3.7. Evidence of Immune Escape Resulting from a Single Mutation in the Globular Head of the F Protein Indirectly Affecting H Protein Epitopes

#### 3.7.1. Escape Mutant Halle-CD46 1.2

**Figure 5:**
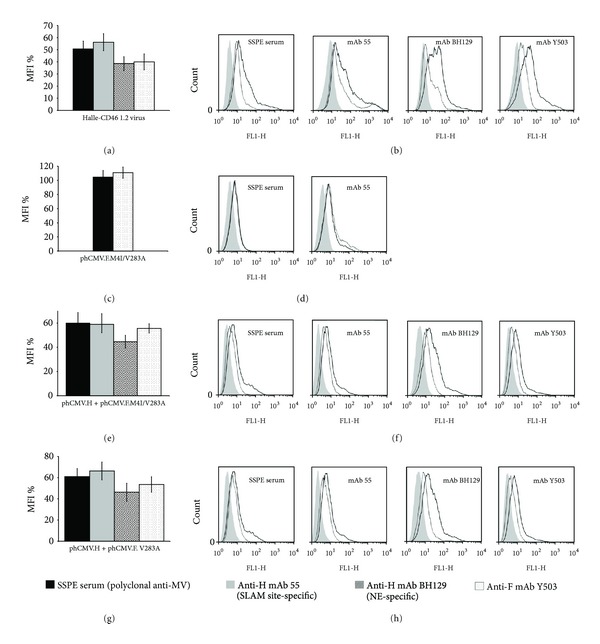
Flow cytometry analysis of escape mutant Halle-CD46 1.2 (H: no change; F: M4I, V283A; M: No change). Vero-SLAM cells infected with the escape mutant virus or transfected with phCMV plasmid expressing the mutated F protein, either alone or with a phCMV plasmid expressing the nonmutated H protein, in the presence of antifusion tri-peptide FIP, were analysed 1 day afterinfection (or after transfection) using the indicated antibodies. Each overlay histogram represents one experiment; the MFI histogram data represent the mean percentages ± standard deviations for three experiments. These values were obtained by using nonmutated Halle virus or expressed nonmutated Halle proteins as controls. (a) and (b)) Escape mutant Halle-CD46 1.2-infected cells; (c) and (d)) cells transfected with phCMV.F.M4I/V283A; ((e) and (f)) and ((g) and (h)) cells transfected with phCMV.H + phCMV.F.M4I/V283A or phCMV.F.V283A, respectively.

This CD46-dependant escape mutant has two mutations in the F protein (M4I, V283A) but none in the H protein or the M protein. Cytometric analysis of Vero-SLAM cells infected with this escape mutant revealed important reductions in the relative fluorescence intensity for the interaction with the polyclonal anti-MeV SSPE serum and anti-H mAb 55 (49% and 44%, resp.; *P* < 0.025) and in particular for the anti-H mAb BH129, specific for the NE epitope [20] and anti-F mAb Y503 (61% and 60%, resp.; *P* < 0.001) (Figures 5(a) and 5(b)). To determine whether these results could be reproduced when the F protein was expressed in the absence of the H protein, Vero-SLAM cells were transfected with phCMV.F.M4I/V283A. Surprisingly, the cytometric analysis showed no reduction in the relative fluorescence intensity for the interaction between the mutated F protein and the SSPE serum or the anti-F mAb Y503 (Figures 5(c) and 5(d)). However, when we cotransfected Vero-SLAM cells with phCMV.H + phCMV.F.M4I/V283A, we obtained a result similar to that obtained with the escape mutant virus with similar reductions for the interaction with all four antibodies (Figures 5(e) and 5(f)). As the protein F from this escape mutant has two mutations (M4I and V283A), we studied them separately to determine the role of each. We found that only the combination phCMV.H + phCMV.F.V283A could replicate the result obtained with the double mutant (Figures 5(g) and 5(h)). Moreover, the biotinylation/western blot assay demonstrated that the mutated F protein and nonmutated H protein in this escape mutant are well expressed at the cell surface (Figure 2(A)-c).

These results can be explained in terms of the reciprocal interaction between the H and F proteins [1]. We speculate that, as a consequence of the physical association between the two glycoproteins, the V283A mutation in the F protein modifies the 3D conformation of the H protein (and thereby its epitopes) which in turn modifies the 3D conformation of the F protein (and mAb Y503's epitope).

### 3.8. Evidence That Mutations in the MeV-M Protein in Combination with Mutations in Either the H Protein Or the F Protein Can Allow Immune Escape

#### 3.8.1. Escape Mutant Halle-CD46 3.3

**Figure 6:**
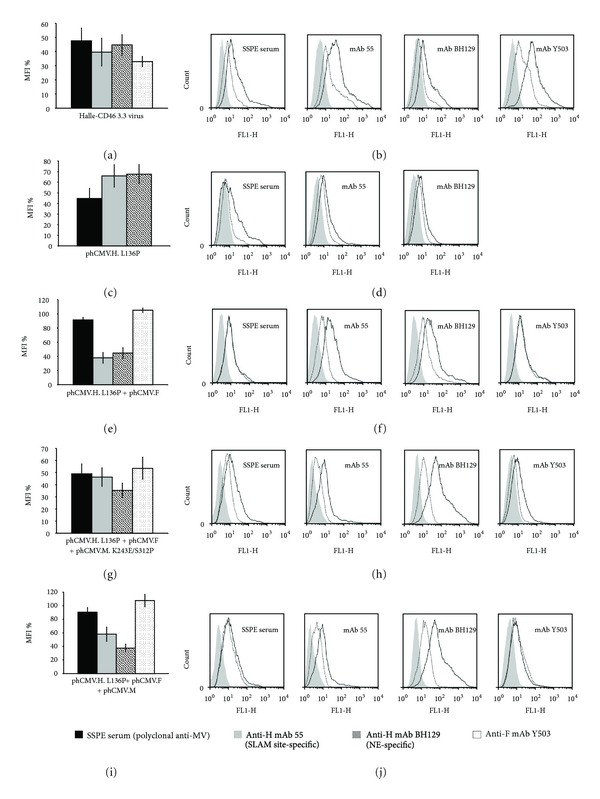
Flow cytometry analysis of escape mutant Halle-CD46 3.3 (H: L136P; F: no change; M: K243E, S312P). Vero-SLAM cells infected with the escape mutant virus or transfected with various phCMV plasmids in the presence, when necessary, of the antifusion tri-peptide FIP, were analysed 1 day after infection (or after transfection) using the indicated antibodies. Each overlay histogram represents one experiment; the MFI histogram data represent the mean percentages ± standard deviations for three experiments. These values were obtained by using nonmutated Halle virus or expressed nonmutated Halle proteins as controls. ((a) and (b)) Escape mutant Halle-CD46 3.3-infected cells; ((c) and (d)) cells transfected with phCMV.H.L136P; ((e) and (f)) cells cotransfected with phCMV.H.L136P + phCMV.F; ((g) and (h)) and ((i) and (j)) cells cotransfected with phCMV.H.L136P + phCMV.F + phCMV.M.K243E/S312P or phCMV.M, respectively.

This CD46-dependant escape mutant has one mutation in the H protein (L136P) that localizes to the stalk region of the protein, two in the M protein (K243E, S312P), but none in the F protein. For the Vero-SLAM cells infected by this escape mutant, the cytometric analysis revealed important reductions in the interaction with all four sera used, in particular for the anti-F mAb Y503 (52%, 60%, 55%, 67% for SSPE serum, mAB 55, mAb BH129, and mAb Y503, resp.; *P* < 0.001; Figures 6(a) and 6(b)). When the H protein from this escape mutant was expressed (phCMV.H.L136P), there was a significant decrease in the interaction with the SSPE serum (55%; *P* < 0.001; Figures 6(c) and 6(d)), but the reduction for the interaction with anti-H mAbs 55 and BH129 was less for phCMV.H.L136P (34% and 32%, resp.; *P* < 0.05) than for the escape mutant virus. Interestingly, a large reduction in the relative fluorescence intensity was observed with the anti-F mAb Y503 despite no mutations being present in the F protein. Moreover, when we cotransfected phCMV plasmids expressing the mutated H with the F protein, the cytometric analysis revealed the absence of a shift with the anti-F mAb Y503 but a large reduction in the relative fluorescence intensity with both anti-H mAbs 55 and BH129 (62% and 55%, resp.; Figures 6(e) and 6(f)). Furthermore, the important negative shift previously observed with the interaction of the mutated H protein with SSPE serum (Figures 6(c) and 6(d)) was not reproduced (Figures 6(e) and 6(f)).

To investigate whether the mutations in the M protein were playing a role, we cotransfected cells with phCMV plasmids expressing the mutated H protein + the F protein + the mutated M protein. The cytometric analysis of this co-transfection revealed important negative shifts with all four sera (51%, 54%, 65%, and 46% for the SSPE serum, mAb 55, mAb BH129, and mAb Y503, resp.; Figures 6(g) and 6(h)). However, when we cotransfected cells with phCMV plasmids expressing the mutated H protein + the F protein + the (nonmutated) M protein, there was no longer a shift with mAb Y503 (Figures 6(i) and 6(j)). Moreover, no such shifts were obtained when cells were cotransfected with phCMV plasmids expressing the H protein + the F protein + the mutated M protein (data not shown). Taken together, these results strongly suggest that the shift observed with Y503 is due to the L136P mutation in the stem of the H protein acting in concert with the mutations in the M protein. As there are two mutations in the M protein (K243E and S312P), we next studied their individual contribution. These cotransfection studies revealed that a shift for mAb Y503 was not obtained when the mutations were present separately in the M protein (data not shown) indicating that the two M protein mutations act in concert to modify the interaction H-F-M with the result that the native conformation of both glycoproteins and hence their epitopes is perturbed. Interestingly, the M protein mutation S312P is present in a SSPE case (UK87/69; accession number: AF503526).

The biotinylation/western blot assay demonstrated that the nonmutated F protein and the mutated H protein in this escape mutant are well expressed at the cell surface (Figure 2(A)-e). A similar result was obtained for the mutated H protein expressed from plasmid phCMV (Figure 2(B)-d). Confocal microscopy confirmed that the mutated M protein, expressed from plasmid phCMV, localizes to the inner plasma membrane beneath them: in Figure 8(b), blue peaks corresponding to the mutated M protein can be seen to colocalize with the green and red peaks corresponding, respectively, to the H and F glycoproteins.

#### 3.8.2. Escape Mutant Halle-SLAM 5.1

**Figure 7:**
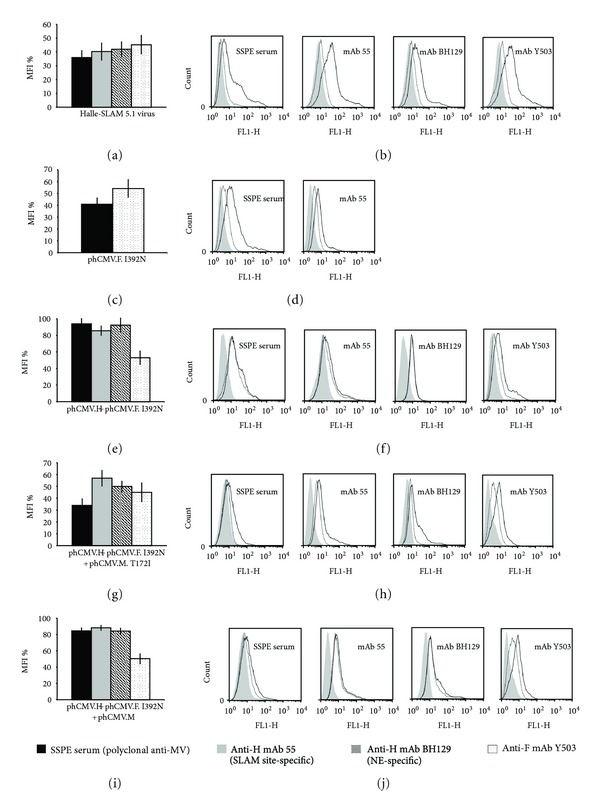
Flow cytometry analysis of escape mutant Halle-SLAM 5.1 (H: no change; F: I329N; M: T172I). Vero-SLAM cells infected with the escape mutant virus or transfected with various phCMV plasmids in the presence, when necessary, of the antifusion tri-peptide FIP were analysed 1 day after infection (or after transfection) using the indicated antibodies. Each overlay histogram represents one experiment; the MFI histogram data represent the mean percentages ± standard deviations for three experiments. These values were obtained by using nonmutated Halle virus or expressed nonmutated Halle proteins as controls. (a) and (b) Cells infected with escape mutant Halle-SLAM 5.1; (c) and (d) cells transfected with phCMV.F.I329N; ((e) and (f)) cells cotransfected with phCMV.F.I329N + phCMV.H; ((g) and (h)) and ((i) and (j)), cells cotransfected with phCMV.F.I329N + phCMV.H + phCMV.M.T172I or phCMV.M, respectively.

This SLAM-dependant escape mutant has one mutation in the F protein (I329N), one in the M protein (T172I), but none in the H protein. The cytometric analysis of cells infected with this escape mutant revealed important reductions in the interaction with all four sera used (64%, 60%, 58%, and 55% for the SSPE serum, mAb 55, mAb BH129, and mAb Y503, resp.; *P* < 0.001; Figures 7(a) and 7(b)), the largest reduction being with the SSPE serum. Transfection of Vero-SLAM cells with phCMV.F.I329N gave similar results for the SSPE serum and Y503 (59% and 46%, resp.; *P* < 0.001) indicating that the single mutation in F is responsible for these negative shifts (Figures 7(c) and 7(d)). To determine why there is a shift with anti-H mAbs 55 and BH129 when there are no mutations in this protein, we transfected phCMV.H with phCMV.F.T172I. This time, the cytometric analysis showed a shift only with anti-F mAb Y503 (47%; Figures 7(e) and 7(f)). As this implicates the mutated M protein, we transfected cells with phCMV.H + phCMV.F.I329N + phCMV.MT173I. This time, the cytometric analysis revealed important negative shifts with all four sera in particular for the SSPE serum (66%, 43%, 50%, and 55% for the SSPE serum, mAb 55, mAb BH129, and mAb Y503, resp.; *P* < 0.001; Figures 7(g) and 7(h)). To determine whether the mutation in the M protein indeed acts in concert with the mutation in the F protein, we cotransfected cells with phCMV plasmids expressing the H protein + the mutated F protein + the (nonmutated) M protein rather than the mutated M protein. This time the only important negative shift was with mAb Y503 (50%; Figure 7(i); compare with Figure 7(g)). Additionally, we cotransfected cells with phCMV plasmids expressing the H protein + the F protein + the mutated M protein. Cytometric analysis of this co-transfection did not give such shifts with any of the four antibodies (data not shown).

**Figure 8:**
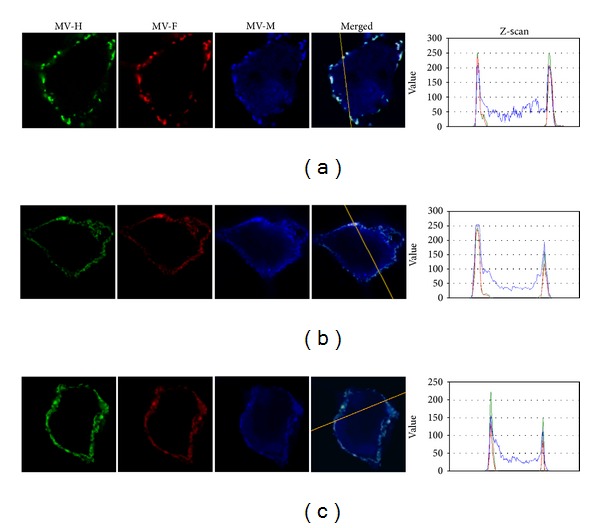
Confocal study of localization of H, F, and M proteins of escape mutants Halle-CD46 3.3 and Halle-SLAM 5.1: (a) phCMV.H + phCMV.F + phCMV.M (control); (b) phCMV.H.L136P + phCMV.F + phCMV.M.K243E/S312P; (c) phCMV.H + phCMV.F.I329N + phCMV.M.T172I. The H, F, and M proteins are labeled green, red, and blue, respectively. Magnification × 630.

The biotinylation/western blot assay demonstrated that the mutated F protein and nonmutated H protein in this escape mutant are well expressed at the cell surface (Figure 2(A)-h). A similar result was obtained for the mutated F protein expressed from plasmid phCMV (Figure 2(C)-c). Confocal microscopy was then made to confirm that the mutated M protein expressed from plasmid phCMV localizes to the inner plasma membrane beneath them: blue peaks corresponding to the mutated M protein can be seen to colocalize with the green and red peaks corresponding, respectively, to the H and F glycoproteins (Figure 8(c)).

We interpret these results in terms of the I329N mutation in the F protein acting in concert with the M protein's T172I mutation to induce epitope-perturbing conformational changes in the F protein, that, due to the glycoproteins' physical association, affect in turn the native conformation of the H protein and its epitopes, thereby allowing immune escape. Importantly, our results suggest that due to the M protein's interaction with the cytoplasmic tails of the glycoproteins, mutations in this protein can potentially influence the conformational state of both the H and F proteins. It should be noted that the protein M mutation T172I is present in two of our escape mutants. Moreover, T172S is present in a SSPE case (Zagreb.CRO/47.02; accession number: DQ227318).

## 4. Discussion

**Figure 9:**
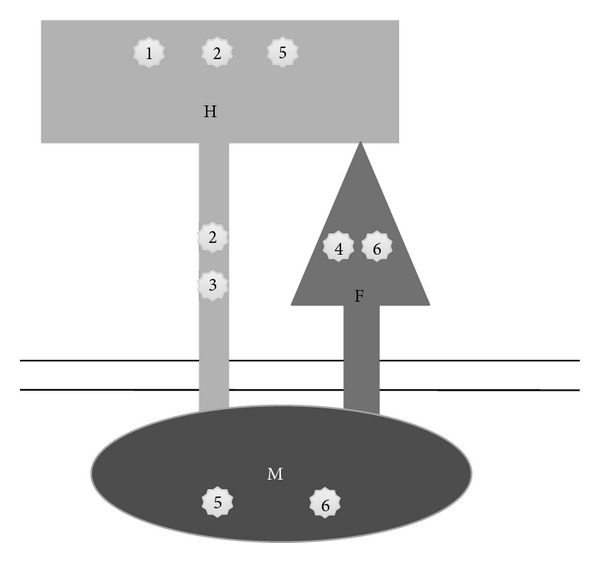
Schematic representation of the MV hemagglutinin (H), fusion (F), and matrix (M) proteins. The localization of the mutations responsible for the immune escape for each of the mutants (numbers1–6) dissected is indicated by the mutant's numbers.

We, and others, have made previous studies examining neutralizing antibody escape in the MV-H protein [10, 12, 19, 26]. Our current results support the finding that the anti-MV humoral response is primarily directed against this glycoprotein [9] but reveal that mutations allowing MV to resist neutralization by anti-MV antibodies are not necessarily located in the major MV-H epitopes. As the analysis of our “minimal” mutants has shown, escape appears to be essentially dependant on conformational changes occurring in the H protein which compromise the recognition of its major epitopes, NE and Noose. Moreover, our results show that there are many ways in which epitopes on this protein can be affected. Most of the mutations we have identified have their effect at distance: mutations affecting the conformation of the H globular head, and hence its epitopes have been found not only in the globular head itself but also in the stalk of the H protein, in the F protein, and even in the M protein (summarized in Figure 9).

That mutations in other domains of the H protein can have an effect on the conformation of this protein's major epitopes and hence their recognition by antibodies is not surprising. Indeed, previously we have shown that the addition of an extra glycosylation site—created by mutating a residue outside of the two major MV-H epitopes—increases escape from polyclonal MV-positive human serum [10]. In addition, we also found that incubation of MV with a cocktail of known MV-H-specific mAbs resulted in several mutations in the H protein that allowed MV to escape neutralization by the mAbs present in the selection cocktail but, importantly, not neutralization by human serum from MV-vaccinated healthy donors [12].

That mutations in the F protein can have an effect on the conformation of the H protein and its epitopes and vice versa can be explained on the basis of the physical interaction between the two glycoproteins. It is believed that, for fusion to occur, structural changes, occurring in the MV-H globular head following receptor attachment, are transmitted to the F protein via the H's stalk domain, which is believed to interact directly with the F protein [27, 28]. Presumably, the F can also influence the H's conformation—again via the stalk domain.

That mutations in the M protein can have an “inside-out” type of effect on the conformation of the glycoprotein ectodomains would appear at first to be surprising but previous studies found that alterations in one glycoprotein's cytoplasmic tail gave advanced cell-cell fusion while double-tail mutants gave even more cell-cell fusion [2]. Moreover, a recombinant matrix-less MV gave extensive cell fusion [3]. On the basis of their results, these authors proposed that, by interacting with the cytoplasmic tails of the glycoproteins, the M protein controls MV fusogenicity—presumably by holding the two glycoproteins in a particular conformational state. Our results thus reinforce the idea of a dynamic physical interaction between these three proteins.

Although generated *in vitro* rather than *in vivo*, it is interesting that both our wt and vaccine strain mutants have similar mutations to those found in SSPE. This introduces the possibility that vaccine strains of MV do not cause SSPE simply because they are less virulent than wt MV strains. SSPE, first described by Dawson in 1933 [29], is a rare fatal sequela of wild-type MV infection where the virus is found persisting in the human brain 8–10 years after an apparently banal acute infection. The mechanism responsible for the pathogenesis of SSPE is still unknown but it has been proposed that anti-MV antibody plays a role in the persistence of the virus [30, 31]. Our results appear to support this hypothesis. It would perhaps be interesting to make similar dissections of the mutations found in SSPE cases to determine whether they contain particular mutations in the H, F, or M proteins of MV that have an effect at distance on the conformation of MV-H epitopes.

It is of interest that cases of SSPE have been reported in vaccinated individuals and that this is due to subsequent infection by a wt virus rather than the vaccine strain [32]. If vaccinated individuals nominally protected by anti-MV antibody are susceptible to wt MV strains, this raises concerns not only for neurological complications of MV but also for its global eradication. That wild-type MV can also accept mutations that do not compromise receptor recognition but allow immune escape underlines the importance of maintaining the monitoring of new emerging strains of the virus.

## Figures and Tables

**Table 1:** Mutations present in the H, F, and M genes of MV-G954 escape mutants obtained by *in vitro* immune selection.

Escape mutant	MV-H mutations	MV-F mutations	MV-M mutations
G-954 without serum	No change	No change	No change
G-954 + serum 1 clone 1	I32V, F111L, I219M	G26S, V107I, H141R, V178A, S285G, Q359P, **E481G**	K92E, **Y114C**, S133G, N168D, **V192M**, **F217L**
G-954 + serum 1 clone 2	K185E, M222T, V322A, D442N, V525A	N380D	**V37A**, N168D, P314L
G-954 + serum 2 clone 1	D135G, **Y252H**, Q334R, C381R	I232V, N392S, **E481G**	S17P, I319T
G-954 + serum 2 clone 2	No change	P227S, R301L, **V550A**	S17P
G-954 + serum 2 clone 3	S335P	M15I	S17P
G-954 + serum 3 clone 1	**R62W**, V91A, S340P, G356S, **E379G**, M602V	P103S, I298V, G376R	S17P, **F50S**, **L122H**, **F276L**
G-954 + serum 3 clone 2	W336R, G591S	No change	S17P
G-954 + serum 3 clone 3	**N200S**, L470S	Q182R, R439W	R86K, N136S, E89K,**V183A**, R225G
G-954 + serum 4	T370A, L454P, T595A	**N186Y**, V322A, **D461G**	S17P, **M51V**, **M53V**, L90F, R297G
G-954 + serum 5 clone 1	M333V, E422G	L68P, S197P, I418T	**Premature termination codon** at amino acid W12
G-954 + serum 5 clone 2	N26S, D404G, E611G. **There are three additional amino acids**: QGC	I36V	S17P, **F54L**, E96G
G-954 + serum 6	H495Y	**F14L**, M49T, G467E	S17P, N208D, **premature termination codon** at amino acid K238
G-954 + SSPE serum clone 1	**I50V**, **H61R**, D128N, **Y232H**, D283N	M15K, A96T, V283A	S17P
G-954 + SSPE serum clone 2	**V485A**	No change	S17P

Escape mutants were selected using Vero-SLAM cells and their H, F, and M genes were then sequenced. The numbers 1–6 refer to the different sera from healthy MV-vaccinated donors used for selection; SSPE refers to the SSPE serum. Escape mutant mutations also found in SSPE cases are in bold. NB: for the G-954 control, two selection steps to isolate G-954 clones were made without sera. Three G-954 clones were isolated and sequencing showed no mutations in their H, F, or M genes. Strikingly, 12 of the 14 escape mutants (86%) contain at least one “SSPE mutation”; that is, a mutation that has also been found in an SSPE case. 42% of the H proteins, 57% of the M proteins, and 36% of the F proteins from the escape mutants contain at least one “SSPE mutation.”

**Table 2:** Mutations present in the H, F, and M genes of MV-Halle escape mutants obtained by *in vitro* immune selection.

Escape mutant	MV-H mutations	MV-F mutations	MV-M mutations	Shift
Normal Halle	No change	No change	No change	control
Halle-SLAM 1.1	**I473M**	M4V, S255G, N408S	No change	+
→ Halle-SLAM 1.2	D332G, T380I	No change	**Y114H**, I319T	+
Halle-SLAM 2.1	I25V, **R62Q**, V259G, **V604A**	D258G, D412G, **L538G**	No change	+
Halle-SLAM 2.2	D90G, **M163T**, **F382S**	**I13M**, I309K, C423R	R36G	+
→ Halle-SLAM 2.3	A158V	No change	M239I	+
Halle-SLAM 3	**K13E**	M4V, F116L, S229P, N380A	**V80A**, T95A	−
Halle-SLAM 4.1	L585P	I216V, K491R, **A515T**	**F73L**, Q310H	+
Halle-SLAM 4.2	L246S, Q311R	No change	Q310H	+
Halle-SLAM 4.3	M45L, **I50F**, **K147E**	No change	No change	−
Halle-SLAM 4.4	**F382S**	No change	Q310H	−
→ Halle-SLAM 5.1	No change	I329N	**T172I**	+
Halle-SLAM 5.2	No change	No change	**V173I**	−
Halle-SLAM 5.3	K364T, W472R	Q133R	K86R, **T172I**	+
Halle-SLAM 6	No change	T543K	No change	−
Halle-SLAM SSPE	H86R, R261G, **R348G**, D507G	S34P, I286V, Y440H, I511V	G245A	+
Halle-CD46 1.1	T93S, A182T, S244P	No change	No change	+
→ Halle-CD46 1.2	No change	M4I, V283A	No change	+
→ Halle-CD46 1.3	**T177I**, R533G	No change	I69T, I196V	+
Halle-CD46 1.4	H17R, **K295E**	No change	D290G	−
Halle-CD46 2.1	T469A	**Y401H**, R439G	**L291I** Premature codon stop at R299	+
Halle-CD46 2.2	No change	L263S, K399R	R181S, L295P	+
Halle-CD46 2.3	K477R	N95S, **R168G**, **N187D**, Y417H	No change	+
Halle-CD46 2.4	Y410C	A124V, R154S, T214S	**L178P**, **F317L**	+
Halle-CD46 3.1	R22G, F180L	S2N, **G40R**, I90F, Q142R, Y254H, F332Y	**I200M**	−
Halle-CD46 3.2	N26I	F332Y	No change	−
→ Halle-CD46 3.3	L136P	No change	K243E, **S312P**	+
Halle-CD46 4.1	R556K	N525S	No change	−
Halle-CD46 4.2	S119A, I407V, S429L, V539A	L68P, I167T, I329V, Q359R	**L280P**, **L291P**, **L294S**, **V303A**	+
Halle-CD46 4.3	No change	No change	N136S, S257N	−
Halle-CD46 5.1	V317D, K375E	V428A, T545I	No change	+
Halle-CD46 5.2	**Y66C**, S73R	Q434L, E458G, **K539E**	**Y5N**	+
Halle-CD46 5.3	No change	No change	D39G, **I260T**	−
Halle-CD46 5.4	**M288K**, S409T	V86A, R102G, I470T, **L553P**	No change	+
Halle-CD46 (6)	D135N, **S169P**	R51S, P319L, N331S	No change	+
Halle-CD46 SSPE 1	S48N, **I50T**, I55F, N262Y, L276F, S285G, **I346V**, **H448R**, K460R, E471A, A496T	R115G, L457W	R30G Premature codon** stop **at R102	+
Halle-CD46 SSPE 2	N77S, **M163T**, G196D	S2T, A129V, M354T	L107P, **Y232H**, **V303I**	+
Halle-CD46 SSPE 3	Q391R	V74I, G256R, V318A, L457W, L507F	Q34R, E93G	+
Halle-CD46 SSPE 4	**Q4L**, L205Q, S532P, D574G	L457W	Q34R	+
Halle-CD46 SSPE 5	L95P, I118T, I427T I435T	**L21H**, V181A, S437G	Q34R	+
Halle-CD46 SSPE 1.1	E161G, **M163T**	I164V, A240V	P118S, V231A, **I302V**	+
Halle-CD46 SSPE 1.2	**K13R**, V80A, **M163T**	I164V, A240G, R535G	No change	+
Halle-CD46 SSPE 1.3	**M163T**, **R195S**, S244P, **F382L**, F571L	V39A, R102G, I164V, A240V	No change	+

CD46-dependant escape mutants were selected using Vero cells and SLAM-dependant escape mutants with CHO-SLAM cells and their H, F, and M genes were then sequenced. The numbers 1–6 refer to the different sera from healthy MV-vaccinated donors used for selection; SSPE refers to the SSPE serum. Escape mutant mutations also found in SSPE cases are in bold. The “Shift” column refers to flow cytometry studies made on the mutants. The Mean Fluorescence Intensity (MFI) values of the mutants were compared with that of Halle (non-mutated control). A plus sign in the “shift” column indicates those mutants having the capacity to induce a statistically important reduction (shift) in the MFI in interaction with any of the sera or specific anti-MV antibodies. Arrows indicate the six escape mutants with a minimal number of mutations that were selected for dissection studies. NB: for the Halle control, two selection steps to isolate Halle clones were made without sera. Three Halle clones were isolated and sequencing showed no mutations in their H, F, or M genes.

**Table 3:** Fusion capacities of the six escape mutants selected for further study relative to Halle.

Virus	Mutations	% fusion
Halle (control)		100
Halle-SLAM 1.2	H.D332G; H.T380I; M.Y114H; M.I319T	84
Halle-SLAM 2.3	H.A158V; M.M239I	180
Halle-SLAM 5.1	F.I329N; M.T172I	69
Halle-CD46 1.2	F.M4I; F.V283A	75
Halle-CD46 1.3	H.T177I; H.R533G; M.I69T; M.I196V	66
Halle-CD46 3.3	H.L136P; M.K243E; M.S312P	95

To study fusion, cells (either CD46-expressing or SLAM-expressing according to the mutant tested) were infected by the different escape mutants and the percentage fusion quantified relative to (non-mutated) Halle (set at 100%).

**Table 4:** Transient neutralization study of the six escape mutants selected for further study.

Virus	Serum 1	Serum 2	Serum 3	Serum 5	SSPE serum
Halle (+ve control)	1/80	1/80	1/400	1/40	1/2400
Halle-SLAM 1.2	1/20				1/600
Halle-SLAM 2.3		1/20			1/1200
Halle-SLAM 5.1				1/20	1/600
Halle-CD46 1.2	1/10				1/600
Halle-CD46 1.3	1/20				1/600
Halle-CD46 3.3			1/50		1/600

The standard Plaque Reduction Neutralization Test (PRNT_50_) was used to give a measure of the neutralizing capacities of anti-MV sera regarding the different mutants. The neutralizing capacity of four sera (sera 1, 2, 3, and 5) obtained from persons immunized against measles virus and one serum from a SSPE patient was studied for the different mutants relative to (non-mutated) Halle. The PRNT_50_ values obtained for each virus are indicated.
